# A comment on “Measuring fractality” by Stadnitski (2012)

**DOI:** 10.3389/fphys.2014.00028

**Published:** 2014-02-05

**Authors:** Maarten L. Wijnants

**Affiliations:** Behavioural Science Institute, Radboud University NijmegenNijmegen, Netherlands

**Keywords:** 1/f noise, fractal, ARFIMA, response time, long memory, cognitive science

In a recent publication Stadnitski (2012) presented an overview of methods to estimate fractal scaling in time series, outlined as an accessible tutorial^1^. The publication was set-up as a comparison between monofractal and ARFIMA methods, and promotes ARFIMA to distinguish between *spurious* and *genuine* 1/f noise, shedding light on “the problem that the log–log power spectrum of short-memory ARMA (*p*, *q*) processes can resemble the spectrum of 1/*f* noise.”

Stadnitski proposes an analytic strategy that consists of fitting 18 models to any time series. Nine of the models are ARMA (*p*, *q*) models, with *p* and *q* varying from 0 to 2, that do not contain long-range correlations. The remaining ARFIMA (*p*, *d*, *q*) models add to the ARMA models a fractional integration parameter *d*. As laid-out by the author, given a genuine fractal series, ARFIMA models should present a better fit than ARMA counterparts. Based on this logic, Stadnitski (2012) evaluates one simple reaction time (SRT) series, and concludes it is not a genuine fractal signal. This conclusion is intriguing, because it was previously argued that SRT series typically present genuine 1/*f* scaling (Van Orden et al., 2003; Wagenmakers et al., 2004).

In this commentary, iteratively refined spectral surrogates (Schreiber and Schmitz, 1996) were generated from the Van Orden et al. (2003) SRT series. This procedure is known to follow the original spectrum more closely than alternative procedures. Next, the performance of a monofractal method (DFA) was compared with the performance of ARFIMA modeling. Using the R-code made available in the Stadnitski tutorial, it is evaluated whether monofractal methods are indeed “distinctly inferior” to the ARFIMA method.

AIC and BIC for nine possible ARMA models and nine ARFIMA models were calculated (see Table 1). For the ten surrogate series, AIC showed best fits for the ARMA (2,0,0), ARMA (0,0,2), ARFIMA (1, *d*, 0) and (2, *d*, 2) model once, and six times for the ARFIMA (0, *d*, 0) model. BIC favored the ARMA (1,0,0) and (2,0,0) model and the ARFIMA (1,d,0) model once, and the ARFIMA (0, *d*, 0) model seven times. Given that “the smallest AIC or BIC indicates the best model,” the *d* parameter was estimated for the best fitting models (for the favored ARMA models, *d* = 0). Next, the 10 surrogate series were analyzed using DFA (converted to *d*), to allow for a comparison between the ARFIMA and monofractal methodologies^2^.

**Table 1:** **Values of the information criteria AIC and BIC for the surrogate time series are shown at the left-hand side. The targeted scaling exponent and the estimated scaling exponents from the various methods are shown on the right-hand side**.

**ARMA**			**ARFIMA**			**d Outcomes**				
**Fitted model**	**AIC**	**BIC**	**Fitted model**	**AIC**	**BIC**	**True d**	**d AIC**	**d BIC**	**d PSD**	**d DFA**
**SERIES 1**										
						0.226	0.15	0.15	0.241	0.154
(0,0,0)	14561.09	14570.96	(0,d,0)	**14516.33**	**14526.19**					
(1,0,0)	14528.59	14543.38	(1,d,0)	14519.06	14533.86					
(0,0,1)	14531.19	14545.98	(0,d,1)	14518.89	14533.68					
(1,0,1)	14522.19	14541.92	(1,d,1)	14516.73	14536.46					
(2,0,1)	14521.76	14546.42	(2,d,1)	14517.84	14542.5					
(1,0,2)	14521.32	14545.97	(1,d,2)	14518	14542.66					
(2,0,0)	14528.79	14548.52	(2,d,0)	14519.05	14538.77					
(0,0,2)	14531.7	14551.43	(0,d,2)	14518.71	14538.43					
(2,0,2)	14521.15	14550.74	(2,d,2)	14518.58	14548.17					
**SERIES 2**										
						0.478	0	0	0.504	0.204
(0,0,0)	14561.09	14570.96	(0,d,0)	14436.47	14446.33					
(1,0,0)	14434.24	14449.04	(1,d,0)	14430.86	14445.65					
(0,0,1)	14463.79	14478.59	(0,d,1)	14433.65	14448.44					
(1,0,1)	14425.31	14445.03	(1,d,1)	14426.87	14446.59					
(2,0,1)	14426.83	14451.48	(2,d,1)	14427.85	14452.51					
(1,0,2)	14426.85	14451.5	(1,d,2)	14427.74	14452.4					
(2,0,0)	**14424.88**	**14444.6**	(2,d,0)	14425.93	14445.66					
(0,0,2)	14434.92	14454.64	(0,d,2)	14428.44	14448.17					
(2,0,2)	14428.83	14458.42	(2,d,2)	14429.73	14459.32					
**SERIES 3**										
						0.338	0.153	0.153	0.314	0.218
(0,0,0)	14561.09	14570.96	(0,d,0)	**14510.44**	**14520.3**					
(1,0,0)	14530.42	14545.21	(1,d,0)	14512.77	14527.56					
(0,0,1)	14534.36	14549.15	(0,d,1)	14512.79	14527.58					
(1,0,1)	14516.42	14536.15	(1,d,1)	14510.71	14530.44					
(2,0,1)	14516.52	14541.17	(2,d,1)	14512.3	14536.96					
(1,0,2)	14516.42	14541.08	(1,d,2)	14512.56	14537.22					
(2,0,0)	14527.45	14547.17	(2,d,0)	14512.55	14532.28					
(0,0,2)	14531.68	14551.41	(0,d,2)	14512.56	14532.29					
(2,0,2)	14512.73	14542.32	(2,d,2)	14513.16	14542.75					
**SERIES 4**										
						0.263	0.149	0.149	0.24	0.18
(0,0,0)	14561.09	14570.96	(0,d,0)	**14518.19**	**14528.05**					
(1,0,0)	14528.69	14543.48	(1,d,0)	14521.33	14536.13					
(0,0,1)	14533.15	14547.95	(0,d,1)	14521.3	14536.09					
(1,0,1)	14524.05	14543.77	(1,d,1)	14522.4	14542.12					
(2,0,1)	14523.33	14547.99	(2,d,1)	14523.65	14548.31					
(1,0,2)	14524.26	14548.92	(1,d,2)	14523.62	14548.28					
(2,0,0)	14525.9	14545.63	(2,d,0)	14521.99	14541.72					
(0,0,2)	14529.11	14548.84	(0,d,2)	14522.1	14541.82					
(2,0,2)	14527.69	14557.28	(2,d,2)	14525.07	14554.66					
**SERIES 5**										
						0.38	0.171	0.171	0.365	0.191
(0,0,0)	14561.09	14570.96	(0,d,0)	**14503.64**	**14513.5**					
(1,0,0)	14523.15	14537.94	(1,d,0)	14506.61	14521.4					
(0,0,1)	14531.06	14545.85	(0,d,1)	14506.61	14521.4					
(1,0,1)	14503.86	14523.59	(1,d,1)	14505.79	14525.51					
(2,0,1)	14505.32	14529.98	(2,d,1)	14508.33	14532.98					
(1,0,2)	14505.24	14529.9	(1,d,2)	14508.18	14532.84					
(2,0,0)	14510.06	14529.79	(2,d,0)	14507.78	14527.51					
(0,0,2)	14518.74	14538.47	(0,d,2)	14507.79	14527.52					
(2,0,2)	14507.76	14537.35	(2,d,2)	14506.05	14535.64					
**SERIES 6**										
						0.334	0	0	0.316	0.177
(0,0,0)	14561.09	14570.96	(0,d,0)	14519.16	14529.02					
(1,0,0)	14512.48	**14527.27**	(1,d,0)	14513.45	14528.24					
(0,0,1)	14518.93	14533.72	(0,d,1)	14515.5	14530.3					
(1,0,1)	14512.47	14532.2	(1,d,1)	14515.3	14535.03					
(2,0,1)	14513.77	14538.43	(2,d,1)	14514.43	14539.09					
(1,0,2)	14513.38	14538.04	(1,d,2)	14513.72	14538.38					
(2,0,0)	14511.95	14531.68	(2,d,0)	14512.45	14532.18					
(0,0,2)	**14511.83**	14531.56	(0,d,2)	14512.15	14531.87					
(2,0,2)	14514.57	14544.16	(2,d,2)	14513.63	14543.22					
**SERIES 7**										
						0.43	0.094	0.094	0.445	0.252
(0,0,0)	14561.09	14570.96	(0,d,0)	14478.19	14488.05					
(1,0,0)	14473.61	14488.4	(1,d,0)	**14470.6**	**14485.39**					
(0,0,1)	14485.32	14500.12	(0,d,1)	14471.15	14485.94					
(1,0,1)	14473.78	14493.51	(1,d,1)	14472.6	14492.32					
(2,0,1)	14476.11	14500.77	(2,d,1)	14472.86	14497.51					
(1,0,2)	14475.43	14500.08	(1,d,2)	14472.68	14497.34					
(2,0,0)	14473.98	14493.71	(2,d,0)	14470.87	14490.6					
(0,0,2)	14476.14	14495.86	(0,d,2)	14470.73	14490.45					
(2,0,2)	14477.28	14506.86	(2,d,2)	14474.4	14503.99					
**SERIES 8**										
						0.35	0.189	0.173	0.307	0.215
(0,0,0)	14561.09	14570.96	(0,d,0)	14505.98	**14515.84**					
(1,0,0)	14511.98	14526.77	(1,d,0)	14506.92	14521.71					
(0,0,1)	14516.75	14531.55	(0,d,1)	14506.67	14521.47					
(1,0,1)	14510.38	14530.11	(1,d,1)	14508.22	14527.94					
(2,0,1)	14506.39	14531.04	(2,d,1)	14507.61	14532.27					
(1,0,2)	14506.39	14531.04	(1,d,2)	14507.46	14532.12					
(2,0,0)	14512.2	14531.92	(2,d,0)	14508.97	14528.7					
(0,0,2)	14515.31	14535.04	(0,d,2)	14508.96	14528.69					
(2,0,2)	14508.32	14537.91	(2,d,2)	**14504.9**	14534.49					
**SERIES 9**										
						0.417	0.252	0.252	0.408	0.381
(0,0,0)	14561.09	14570.96	(0,d,0)	**14414.71**	**14424.57**					
(1,0,0)	14452.18	14466.97	(1,d,0)	14418.63	14433.43					
(0,0,1)	14475.15	14489.94	(0,d,1)	14418.63	14433.43					
(1,0,1)	14421.86	14441.59	(1,d,1)	14419.1	14438.82					
(2,0,1)	14417.36	14442.01	(2,d,1)	14420.51	14445.17					
(1,0,2)	14417.33	14441.98	(1,d,2)	14420.61	14445.26					
(2,0,0)	14439.83	14459.55	(2,d,0)	14418.71	14438.44					
(0,0,2)	14460.14	14479.86	(0,d,2)	14418.77	14438.49					
(2,0,2)	14419.58	14449.17	(2,d,2)	14417.69	14447.28					
**SERIES 10**										
						0.212	0.128	0.128	0.2	0.188
(0,0,0)	14561.09	14570.96	(0,d,0)	**14529.06**	**14538.92**					
(1,0,0)	14539.54	14554.34	(1,d,0)	14532.72	14547.51					
(0,0,1)	14541.64	14556.44	(0,d,1)	14532.72	14547.52					
(1,0,1)	14533.99	14553.72	(1,d,1)	14533.26	14552.98					
(2,0,1)	14532.51	14557.16	(2,d,1)	14535.2	14559.85					
(1,0,2)	14532.38	14557.03	(1,d,2)	14535.23	14559.89					
(2,0,0)	14539.54	14559.26	(2,d,0)	14533.81	14553.54					
(0,0,2)	14540.86	14560.59	(0,d,2)	14533.75	14553.47					
(2,0,2)	14534.41	14564	(2,d,2)	14533.5	14563.09					

As pointed out by Stadnitski, “good estimators are unbiased, i.e., their means equal the true parameter value.” The summed absolute difference between the estimated *d*-values from the surrogates and the original data was 3.86 for AIC, 3.87 for BIC, and 1.27 for DFA. Hence, DFA equaled the target parameters much more closely. DFA approached the target *d*-values more closely than ARFIMA in ten out of ten cases. Hence, the claim that monofractal methods are inferior to ARFIMA methods is not supported. Contrary, this analysis shows that monofractal methods in fact provide much better estimates.

This conclusion is discrepant to Stadnitski's conclusion. Stadnitski simulated a short-memory ARIMA (1,0,1) model and a long-memory ARFIMA (1, *d*, 1) model, and concluded that ARFIMA methods were less biased and more precise than, and therefore superior to, monofractal estimators. Here, spectral surrogates were constructed, and ARFIMA and DFA estimates were compared to the original target parameters. Here, the ARFIMA methodology was more biased than monofractal estimators. So how should one go about this discrepancy?

Accuracy concerns aside, it may be concluded that the issue raised by Stadnitski is in fact a theoretical, rather than a statistical one. By realizing that “goodness-of-fit alone cannot serve up counterexamples that falsify theories” (Gilden, 2009, p. 1463), it is argued that fitting ARFIMA algorithms to the data is not sufficient in itself to distinguish between genuine and spurious scaling properties. The true challenge should be “to compare the empirical accuracy of theoretical predictions in a program of strong inference” (Hasselman, 2012, p. 4).

That is, although often implicit, there must exist specific ontological intuitions that motivate researchers to fit ARFIMA models. As it stands, however, this inherent theoretical motivation is still to take up the challenge against theoretical predictions corroborated by fractal perspectives (see Diniz et al., 2011), like the ubiquity of 1/f scaling, consistent changes away from 1/f scaling with pathological conditions and aging (Goldberger et al., 2002; Hausdorff, 2007), consistent changes toward 1/f scaling in more skilled performances (Wijnants et al., 2009, 2012a,b), no bending at the low-frequencies in a power spectrum when longer time series are collected (Van Orden et al., 2005), and so forth. In short, spurious 1/f scaling “becomes an extraordinary hypothesis that would itself require extraordinary evidence” (Van Orden et al., 2003, p. 19), evidence that cannot come from goodness-of-fit alone, to convince that the preferred models are also theoretically viable, and consequently to be preferred methodologically.

## Footnotes

^1^In this short commentary, the terminology of the Stadnitski paper (including acronyms and short notations) has been used.

^2^For a full description of the methods, please consult Stadnitski (2012).

## References

[B1a] Diniz A. Wijnants M. L. Torre K. Barreiros J. Crato N. Bosman A. M. T. (2011). Contemporary theories of 1/f noise in motor control. Hum. Mov. Sci. 30, 889–905 10.1016/j.humov.2010.07.006 21196059

[B1] Gilden D. L. (2009). Global model analysis of cognitive variability. Cogn. Sci. 33, 1441–1467 10.1111/j.1551-6709.2009.01060.x 20376202 PMC2850128

[B2] Goldberger A. L. Peng C.-K. Lipsitz L. A. (2002). What is physiologic complexity and how does it change with aging and disease? Neurobiol. Aging 23, 23–26 10.1016/S0197-4580(01)00266-4 11755014

[B3] Hasselman F. (2012). When the blind curve is finite: dimension estimation and model inference based on empirical waveforms. Front. Physiol. 4:75 10.3389/fphys.2013.00075 23580349 PMC3619109

[B4] Hausdorff J. M. (2007). Gait dynamics, fractals and falls: finding meaning in the stride-to-stride fluctuations of human walking. Hum. Mov. Sci. 26, 555–589 10.1016/j.humov.2007.05.003 17618701 PMC2267927

[B5] Schreiber T. Schmitz A. (1996). Improved surrogate data for nonlinearity tests. Phys. Rev. Lett. 77, 635 10.1103/PhysRevLett.77.635 10062864

[B6] Stadnitski T. (2012). Measuring fractality. Front. Physiol. 3:127 10.3389/fphys.2012.00127 22586408 PMC3345945

[B7] Van Orden G. Holden J. G. Turvey M. T. (2003). Self-organization of cognitive performance. J. Exp. Psychol. Gen. 132, 331–350 10.1037/0096-3445.132.3.331 13678372

[B8] Van Orden G. Holden J. G. Turvey M. T. (2005). Human cognition and 1/f scaling. J. Exp. Psychol. Gen. 134, 117–123 10.1037/0096-3445.134.1.117 15702967

[B9] Wagenmakers E.-J. Farrell S. Ratcliff R. (2004). Estimation and interpretation of 1/*f* ^α^ noise in human cognition. Psychon. Bull. Rev. 11, 579–615 10.3758/BF03196615 15581115 PMC1479451

[B10] Wijnants M. L. Bosman A. M. T. Hasselman F. Cox R. F. A. Van Orden G. (2009). 1/f scaling in movement time changes with practice in precision aiming. Nonlinear Dynamics Psychol. Life Sci. 13, 75–94 19061546

[B11] Wijnants M. L. Cox R. F. A. Hasselman F. Bosman A. M. T. Van Orden G. (2012a). A trade-off study revealing nested timescales of constraint. Front. Physiol. 3:116 10.3389/fphys.2012.00116 22654760 PMC3359523

[B12] Wijnants M. L. Hasselman F. Cox R. F. A. Bosman A. M. T. Van Orden G. (2012b). An interaction-dominant perspective on reading fluency and dyslexia. Ann. Dyslexia 62, 100–119 10.1007/s11881-012-0067-3 22460607 PMC3360848

